# Buffalo Academy of Medicine

**Published:** 1901-05

**Authors:** Thomas F. Dwyer


### Society Proceedings.

### BUFFALO ACADEMY OF MEDICINE.

REPORTED BY THOMAS F. DWYER, M. D., Secretary.

THE regular quarterly meeting of the Buffalo Academy of Medicine was held at the Academy rooms, Public Library Building, Tuesday, March 19, 1901. The meeting was called to order at 9 p. m. by the president, Dr. MARCEL HARTWIG.

The minutes of the last quarterly meeting, December 4, 1900, were read and approved.

The nomination of officers resulted as follows: For president, Charles G. Stockton; secretary, Francis W. McGuire; treasurer, Charles S. Jewett; trustees, Arthur W. Hurd, J. Henry Dowd, Matthew D. Mann.

The program of the evening, under the auspices of the section of pathology, consisted of the following: A report of recent investigations of cancer, by H. R. Gaylord; A case of organic epilepsy, clinical and pathological report, by Henry P. Frost.

Attendance, 53. Adjourned at 11 p.m.

---

### *Section on Pathology, February 19, 1901.*

REPORTED BY F. W. McGUIRE, M. D., Secretary.

The regular meeting of the Pathological Section was held at the Academy rooms, Tuesday, February 19, 1901. Present, 18 Fellows. In the absence of Dr. EUGENE A. SMITH, Dr. BENJAMIN G. LONG was elected chairman.

The first essayist of the evening was Dr. CHARLES S. JEWETT who read a paper, entitled,

#### LEUKEMIA.

The doctor gave a brief review of the earlier work of Virchow, Craigie and Bennett, Vogel, Neumann and others, on leukemia, the various theories regarding the nature of this disease were discussed, and it was shown that the theory of Virchow and Neumann, in combination with the modern theory of infection offers the best explanation at present available. In leukemia the specific causative organism has not yet been demonstrated.

Two chief varieties of the disease are now recognized: (1) the myelogenous or splenic-myelogenous form, characterised chiefly by the enormous number of myelocytes found in the circulating blood, also by the presence of eosinophilic myelocytes and of atypical forms and an increase of mast cells and of eosinophiles and the presence of large numbers of nucleated red cells, a form that is always chronic; (2) the lymphatic form, in which is found a great, absolute and relative increase of the lymphocytes. In this form the nucleated red cells are present, but less numerous, and the anemia is usually far more pronounced. Lymphatic leukemia may be either acute or chronic.

The writer then reported three cases of leukemia which had come under his observation, giving in detail the results of the blood examinations.

One was a case of myelogenous leukemia, in a man of 36 years, the others were of the subacute lymphatic variety and both occurred in infants. In the two latter cases, owing to the failure to secure autopsies, it was difficult to differentiate leukemia from the severe secondary anemia of infancy, but the marked lymphemic condition of the blood combined with the absence of all demonstrable cause for a secondary anemia seemed to justify the diagnosis of leukemia.

DISCUSSION.

Dr. Irving P. Lyon was interested in the paper. He thought cases two and three were too young to make a positive diagnosis of leukemia, as the blood of children under one year show a marked leucytosis. He said Van Jaksch anemia pseudo-leukemia infantum was generally due to rickets or syphilis.

Dr. Charles G. Stockton asked the author to explain the connections between pseudo-leukemia and leukemia, as some cases in the beginning resembled each other.

Dr. A. L. Benedict said he would hesitate to make a diagnosis from the examination of the blood alone without a post mortem.

Dr. Charles S. Jewett said that he did not wish to press the issue that the diagnosis of cases two and three were correct, but after several careful examinations of the blood he thought that his diagnosis was the most reasonable.

The next paper was an extemporaneous discussion by Dr. Lyon on

THE DISTRIBUTION OF CANCER IN BUFFALO FOR THE TWENTY YEAR PERIOD, 1880–1899.

The doctor said the purpose of investigation was to ascertain whether the distribution of cancer mortality through the city corresponded with the distribution of population, or showed an irregular distribution with local foci, resembling the endemic foci of certain infectious diseases. The secondary purpose of investigation was to develop any facts whatever that might be of interest in relation to the parasitic theory of cancer.

The city mortality records were examined and all cases dying of malignant growth—carcinoma, sarcoma, epithelioma, malignant tumor and the like, were culled out, numbering 2,413; of these 2,413 cases, as the result of a careful examination of the death certificate, hospital records, city directory, etc., 114 cases were excluded as non-resident, leaving a total of 2,299 cases of malignant tumor accredited to the city. Of the 2,299 cases, the house address of 294 could not be ascertained. These 2,005 cases were plotted on the city map according to residence, a colored dot representing each case. The distribution of cases thus shown, was found to be notable in one respect, viz.: a marked concentration occurred in the German and Polish quarter of the city. The equally populous Italian quarter, centering in Canal Street, showed by contrast a conspicuous absence of any cancer concentration; no relation could be established between cancer distribution and any other condition. The concentration in the German and Polish district was the only noteworthy fact.

The map also indicated the houses that had been the seat of more than one case dying with cancer, the dots representing such cases being surrounded by a circle for each such house. There were 44 such "cancer houses," 41 showing 2 cases, and 3, 3 cases each, making a total of 91 cases in such houses. Thus 91 out of 2,005 cases, or 4.53 per cent., died in "cancer houses." Dr. Lyon did not care to affirm that this percentage was more than was to be expected, but was inclined to think so, considering the fact that the number of dwellings in the city was 37,290 in 1890, and is probably over 50,000 at present.

The death-rate from cancer in Buffalo showed a steady increase, rising from 32 to 53 per 100,000 of population from 1880 to 1899, and thus agreeing with the reports from all civilised countries.

The age distribution agreed with other well known statistics. The race distribution was most important, Germans and Poles constituting 42 per cent. of the cancer mortality of the city, though making only 20 per cent. of the population (U. S. Census, 1890).

This fact agreed with the map concentration in the German and Polish section of the city, previously mentioned. Those of native (U. S.) birth constituted only 29 per cent. of the cancer mortality, as aganist 65 per cent. of the population of the city. Those of foreign birth in general showed a cancer mortality out of proportion to their population, as the Germans and Poles constituted 57 per cent. of all the foreigners, and as they inhabit in general a certain section of the city, it is of interest why such a section shows a great cancer concentration as indicated by the map.

The distribution of cancer by organs involved in the different classes of population was most important. Cancer of the stomach occurred in 47 per cent. of all males and 25 per cent. of all females dying of cancer, or 34 per cent. of both sexes. For the Germans and Poles the corresponding figures were 52 per cent., 35 per cent. and 43 per cent., and for those born in the United States 37 per cent., 13 per cent. and 21 per cent.

It is thus seen that the proportion of cancer of the stomach and cancer of all organs is more than twice as great in the Germans and Poles as in those born in the United States. This fact seems of great importance in pointing to the diet as a probable factor in determining the frequence of location of cancer in the stomach. It seems hardly possible to explain the variation between the two classes of population otherwise. Accepting this explanation, then, how can the diet affect this result? The choice of two theories is open. (1) The diet may be supposed to infect the stomach with a cancer producing organism, which organism occurs on certain articles of diet more generally used by the Germans; or (2) it may be supposed that the German abuses his stomach by his food and drink and thus increases its liability to cancer according to the trauma theory or Cohnheim theory. Dr. Lyon was of the opinion that the evidence for the abuse of the German stomach by diet was largely lacking, to support this theory, and that the doubled frequence of attack of the German stomach, as compared with the American stomach was best explained in the theory of direct infection through the diet.

Behla's observations at Luckan, Germany, supported the parasitic theory and laid suspicion upon uncooked vegetables, as the carriers of infection. As is well known the Germans are great eaters of raw vegetables, salads, and the like.

At any rate, whatever the theory of explanation, the important fact remains that the German stomach is remarkably liable to invasion by cancer, a fact apparently not previously known.

Among other interesting facts brought out by Dr. Lyon was the ratio of male to female mortality from cancer for different classes of population, representing as 100 the female mortality; the male mortality for Germans and Poles was 93, for those born in the United States, 51. The Germans and Poles stood in a class apart from all other nationalities in showing a close approximation of female mortality for cancer by male mortality, a marked difference between male and female mortality being shown by all other races. This was apparently explained by the fact that cancer of the breast and uterus was relatively less frequent in the German and Poles than in other races, as indicated by the Buffalo statistics.

Summarising his work, Dr. Lyon said that the statistics of Buffalo, show that the Germans and Poles of the city are 4.81 more susceptible to cancer than those of native birth (U. S.) and that the Germans and Poles in Buffalo are 10.00 times more liable to cancer of the stomach than those born in the United States. The facts are startling and apparently are but explained on the parasitic theory of cancer.

DISCUSSION.

Dr. A. L. Benedict was very much impressed with the number of cases of cancer of stomach in the German population of Buffalo. He thought that the American born should be in the excess as they more frequently abuse their stomachs. He said he had cured a number of cases that had been diagnosticated by others as cancer.

Dr. H. R. Hopkins thought there was a source of error in the house theory of infection. He would like to know what kind of houses they were where cancer had occurred more than once. Whether these houses were cottages, double houses or flats? He thought that some of us would change our minds regarding the American abusing his stomach if we had noticed the character of the food ejected from some of the foreigners' stomachs. He thought that the Americans eat wholesome food as compared with the foreign classes.

Dr. Grover W. Wende said that at least half of the children attending the schools were of German parentage, and offered this circumstance for the author's consideration.

Dr. Charles G. Stockton congratulated the author on the statistics and amount of work he had undertaken. He thought it remarkable that in the German women there was only four cases in the upper tract, as the tongue and jaw, as compared with twenty-seven in the German male. He also said a number of the complaints of the American stomach was only functional and could not agree that the American abused his stomach more so than the German.

Dr. H. R. Gaylord was much interested in Dr. Lyon's work, and knew that the doctor was very careful in compiling his statistics. He said in Germany and London they had formed societies for the investigation of cancer and thought the United States should do the same. The fact that such a man as Metschnikoff who had not eaten raw vegetables for twenty years, believing that cancer was caused by organism on them, lent support to that theory. He also stated that statistics might give us the cause before direct examination would reveal it, and believes that something in the diet to be the cause of cancer.

Dr. Sydney A. Dunham thought that the continued irritation of the stomach in the Germans was probably the cause of so frequent cancer in that race.

Dr. B. G. Long said females live longer than males, as he had made investigations which supported that theory and thought that that fact could account for the greater number of cancer in females. He thought the American stomach was not so much abused as the German.

---

*Section on Obstetrics and Gynecology, February 26, 1901.*

Reported by WM. G. RING, M. D., Secretary.

The regular monthly meeting of this section was held on Tuesday evening, February 26, 1901, at 8.30 p.m Dr. A. J. Colton, presiding.

The minutes of the previous meeting were read and approved.

RUPTURE OF THE UTERUS.

Dr. P. W. Van Peyma reported a case of multiparous delivery; pains were feeble; head presented, later nothing presented. Dr. Schroeter was sent for also Dr. Saylin; patient pulseless; small parts of child was felt under skin; cervix dilated and child delivered; placenta was in abdomen. No cause for rupture of the uterus was discovered. Placenta was delivered through the rent. Woman was doing well, but finally died of peritonitis. Dr. Schroeter gave further details of the same case.

Dr. J. W. Grosvenor asked Dr. Schroeter, if it would not have been well to have opened the abdomen and repaired the rent in uterus. Dr. Schroeter thought not.

Dr. M. D. Mann said that statistics showed that best results followed operation as contrasted with drainage.

Dr. LUDWIG SCHROETER said that the statistics he had looked up gave the reverse results.

Dr. P. W. VAN PEYMA said the cases he had seen—two or three —had been operated on and all died. One case without operation recovered. Dr. Carl Braun, of Vienna, advocated no operation. Operation or not, mortality is very great.

Dr. M. A. CROCKETT read a paper, entitled,

THE TOXEMIAS OF PREGNANCY. (See p. 736.)

Dr. P. W. VAN PEYMA followed by the reading of a paper on

SYMPHYSIOTOMY.

DISCUSSION.

Dr. M. D. MANN—Dr. Crockett has gone over the subject so carefully that it leaves little to add. I think the subject a most important one. Although we do not know what poisons are present, we can know how to get rid of them. I recall one case of nausea and vomiting, with frightful headache; she was obliged to take morphine—two or three grains at a dose—vomiting was continuous. We tried to get kidneys to excrete; the skin was dry and harsh. Emptied the uterus and finally the kidneys worked. Patient was sent to the country; she had headache and was given morphine, following which she had the entire train of symptoms repeated; it was with the greatest difficulty we saved her. I must say that the plan of discussing two papers at once is a poor plan and hope it will be changed.

In regard to Dr. Van Peyma's paper would say that the operation of symphysiotomy has never been done in Buffalo. He had never even seen the operation. It has a field in obstetric practice, though limited. Many cases reported of extra mobility of pelvis. Dr. Ayers urges wiring the bones. If bones were wired with silver wire the bad after-effects would be obviated. Four successful cases are reported. Statistics of Cesarean section show marvelous success; it comes as a distinct rival of the operation under discussion.

Dr. LUDWIG SCHROETER.—The subject of Dr. Crockett's paper is new. One of the points in the paper in reference to the liver is true.

With 12 per cent. mortality, we cannot expect the operation of symphysiotomy will become popular. If patient is saved by Cesarean section, she is saved in perfect health. After symphysiotomy they complain of backache and inability to bend down.

Prof. Fritsch says he never makes a symphysiotomy dependent upon the measurement of the conjugate diameter of the pelvis. If we follow Prenaud's advice we should abandon forceps altogether.

Dr. J. W. Grosvenor.—The use of salt solution is very effective in deficient elimination. I recall a case in which the patient was given one gallon by mouth in twenty-four hours; it was effective in warding off eclampsia. I recommend the hot air bath.

Dr. Geo. N. Jack.—I was greatly interested in Dr. Crockett's paper. I think when physicians study bio-chemistry, tissue metabolism, and similar processes, they will use less drugs. I think symphysiotomy is the coming operation. Kelly reports thirty successful cases.

Dr. S. Y. Howell.—The importance of toxemia during pregnancy cannot be overestimated. We should study our patient during that time. A careful study of the twenty-four hours urine, and of the total solids should be made. Careful attention should be paid to the skin, as it is the great safety-valve of the kidneys. The condition of the bowels is of the utmost importance.

Dr. Edward L. Frost asked what proportion of the albumin found in urine came from the bladder, and cited a case; he also asked if the autointoxication was the result of pregnancy.

Dr. McClellan Pomeroy complimented the essayists, and stated that the whole subject of toxemia was indefinite at the present time, and it was difficult to say what would be the final outcome of its study. He saw one case of symphysiotomy in Montreal, Can. He did not believe the operation would become general. The doctor also recommended the single discussion of papers.

Dr. P. W. Van Peyma, in closing, said that he was much interested in the subject of eclampsia. He felt now, as formerly, that it is a fine field for investigation. Nausea and vomiting as a premonitory symptom is a very good suggestion, although it is not so in my experience.

Dr. M. A. Crockett, in closing, said that Dr. Frost drew correct conclusions; the autoinfection is the result of pregnancy.

Dr Van Peyma added that union after symphysiotomy, made by suture, was open to the objection that it was followed by necrosis of bone.

Dr. Ludwig Schroeter said that the mortality of symphysiotomy was 12 per cent; that is under all circumstances, but not so under favorable ones. Many groups of cases have been reported without a single death. It is true that women cannot do manual labor for a long time after the operation. As to Dr. Fritsch's suggestion, no one source of information can be depended upon.

Meeting adjourned at 11 p.m. Present, 25.

---

*Section on Obstetrics and Gynecology, March 26, 1901.*

REPORTED BY SYDNEY A. DUNHAM, M. D., Secretary pro tem.

The regular meeting of this section was held March 26, 1901, at 8.30 p.m. ALBERT J. COLTON in the chair.

The meeting opened with fourteen members present.

Dr. STEPHEN Y. HOWELL read a paper on

OVARIAN HERNIA.

Dr. LAWRENCE G. HANLEY, who was down for a second paper on the Relation of Pelvic Disease to Insanity, was unable to be present.

Dr. STEPHEN Y. HOWELL reported in his paper a case in which the ovary had invaded the right inguinal canal, on which he operated with recovery. Chief symptoms were pain, due to pressure and also sickening sensation. There was much inflammatory tissue around ovary. A truss had been previously worn. In reviewing the history of some cases, Dr. Howell believed the inguinal variety the most common.

DISCUSSION.

Dr. McCLELLAN POMEROY said that Sajous gave the crural variety the most common, and womb displacement the chief cause.

Dr. E. A. SMITH had found various viscera in the hernial sac, *e. g.*, undescended testes and appendix.

Dr. C. E. CONGDON had found the ovary in the sac of a femoral hernia.

Meeting adjourned at 9.45 p.m.

---

*Section on Medicine, March 12, 1901.*

REPORTED BY JULIUS ULLMAN, M. D., Secretary.

Dr. SIGMUND GOLDBERG, chairman pro tem., presiding.

Dr. JULIUS ULLMAN read a paper on

THE RACIAL FACTOR IN HYSTERIA.

Dr. Ullman before discussing hysteria in the human species cited the reports of M. Aruch, who studied the disease in dogs, and of Hegier, who reported hysteria in a cat and a canary bird. In the genus man, hysteria has been confined to no one time, latitude or race. Though no direct reference is made to it in the Bible, in the light of our knowledge the miracles performed by the Nazarene must be interpreted as hysterical manifestations. The Greeks wrote entertainingly of it. The writer is of the opinion that racial characteristics are not so much the result of inherent qualities as of social conditions, and after classifying the physical and psychological causes he considered the social conditions which increase the disease relatively among various races.

Reference was made to the continued history of persecution among the Jews, their fertile mentality, the conditions of oppression, their exclusion from trades and professions, their isolation by segregation among themselves in ghettoes, their burdening with taxes and the tendencies this gave for consanguineous marriage and the learning to simulate hysteria the child from the mother.

Reference also was made to the increase of hysteria among the Latin and Slavic races, among the later of whom it can be attributed to conditions of surfdom but little better than slavery. Witchcraft and demoniacal possessions were spoken of as built in a ground work of hysteria and superstition, for as Fére says, the hysterical anesthesias hold first place as the marks of the devil. The Salem witchcraft in New England was spoken of, and it is not surprising to the writer that among the progenitors of the believers of witchcraft that other delusory belief of Christian science should have had its inception.

The history of the negro of Africa was mentioned. Their forcible kidnapping and horrifying voyage to this country in the slave trading ships gave sufficient reason for a psychical insult, the results of which may still be seen in the hysterical manifestations of the negro in this country, especially at religious revival meetings.

The history of the native American Indian among whom it was shown that hysteria could not have existed was given, and the effects of civilisation and assimilation on the increase of the disease among them was shown.

The Mongol and Hindoo races were alluded to, among whom it is seen relatively less frequent.

It was therefore shown that hysteria exists among all peoples and in all countries, and the fact that certain races, such as the Jewish, the Slavic, Latin, and possibly the negro are relatively more affected may be attributed to causes of environment rather than inherent qualities, for as Jacobs says, if all the Johns and Marias were to be shut up in ghettoes for a couple of centuries, they would undoubtedly show peculiarities in habit, and thought, and would develop a peculiar psychology. Heredity plays a rôle only in that a child may learn to imitate certain traits of an hysterical parent.

In conclusion, it was emphasised that hysterical symptoms may exist as epiphenomena and a background in organic diseases among susceptible races, or that though symptoms of hysteria are well marked the patient may at the same time have organic disease.

Better therapeutic results will therefore be obtained among those in whom hysteria is relatively more common by the administration of drugs, suggestion and mechanical treatment,' directed aganist a latent hysterical condition in addition to treatment for the organic disease, if it be present.

Dr. Geo. F. Cott read a short paper upon the subject,

HYSTERICAL APHONIA.

He went into the pathology as far as known, and giving the different theories so far advanced as to its causation. He pointed out the fallacy of physicians in general maintaining that the vocal organs are absolutely under the will and control of the patient, since the patient is in reality positively hopeless until proper equilibrium is reëstablished. One point is important to emphasise: in hysterical aphonia both cords are similarly affected always; he, however, cited a curious condition where there was anchylosis of the left arytenoid articulation, leaving the cord fixed near the median line and every effort made by the patient to produce a sound the false cord could be seen closing upon its fellow on the opposite side, and a harsh sound or loud whisper produced that way. This again shows the possible function of the false cords. The paper closed by citing several peculiar and most interesting cases.

Dr. William C. Krauss reported a number of unusual cases of hysteria, one of which was of especial interest. It was a young girl, observed with Dr. Ullman, in whom there were unusual contractures; the hands and fingers were in a condition of flexion, the toes in extension. There also was hyperesthesia, a spasm of the orbicularis palpebrarum and aphonia. The patient became bedridden but was eventually cured by suggestion.

Dr. A. W. Bayliss gave a lantern slide exhibition on the use of

THE *x*-RAY IN MEDICAL AND SURGICAL PRACTICE.

He showed fractures, dislocations, foreign bodies, deformities of bone and joints, as well as tubercular chests, and other interesting cases were demonstrated

Drs. Sydney A. Dunham, Grosvenor and Krauss discussed the subject.

